# BMP-2 Is Involved in Scleral Remodeling in Myopia Development

**DOI:** 10.1371/journal.pone.0125219

**Published:** 2015-05-12

**Authors:** Honghui Li, Dongmei Cui, Feng Zhao, Lijun Huo, Jianmin Hu, Junwen Zeng

**Affiliations:** 1 State Key Laboratory of Ophthalmology, Zhongshan Ophthalmic Center, Sun Yat-sen University, 54 South Xianlie Road, Guangzhou, 510060, P. R. China; 2 The First Affiliated Hospital, Sun Yat-sen University, 58 Zhongshan Second Road, Guangzhou, 510080, P. R. China; 3 The Second Affiliated Hospital of Fujian Medical University, 34 North Zhongshan Road, Quanzhou 362000, P. R. China; Wenzhou Medical University, CHINA

## Abstract

The development of myopia is associated with scleral remodeling, but it is unclear which factors regulate this process. This study investigated bone morphogenetic protein-2 (BMP-2) expression in the sclera of guinea pigs with lens-induced myopia (LIM) and after recovery from myopia and evaluated the effect of BMP-2 on extracellular matrix (ECM) synthesis in human scleral fibroblasts (HSFs) cultured *in vitro*. Lens-induced myopia was brought about in two groups of guinea pigs (the lens-induced myopia and myopia recovery groups) by placing -4.00 D lenses on the right eye for three weeks. The left eye served as a contralateral control. In the recovery group, the lenses were removed after one week. The refractive power and axial length of the eyes were measured, and the BMP-2 expression levels in the sclera were measured. After three weeks, the lens-induced eyes acquired relative myopia in both groups of guinea pigs. Immunostaining of the eyeballs revealed significantly decreased BMP-2 expression in the posterior sclera of the myopic eyes compared to the contralateral eyes. One week after lens removal, BMP-2 expression recovered, and no differences were observed between the experimental and contralateral eyes in the recovery group. HSFs were cultured with BMP-2 or transforming growth factor-β1 (TGF-β1). Type I and type III collagen synthesis was significantly up-regulated following BMP-2 treatment in culture after one and two weeks, but the ratio of type III to type I collagen mRNA was not increased. Biosynthesis of glycosaminoglycan (GAG) and aggrecan was increased in HSFs treated with BMP-2. Some chondrogenesis-associated genes expression increased in HSFs treated with BMP-2. From this study, we concluded that BMP-2 is involved in scleral remodeling in the development and recovery of lens-induced myopia.

## Introduction

Myopia is a highly prevalent ocular disease worldwide[1,2,3,4]. The major structural change in myopia is an excessive increase in axial length[5,6]. This ocular enlargement increases the risks of pathological damage such as macular degeneration, subretinal hemorrhage and retinal detachment, leading to irreversible vision loss and blindness[7,8].

Though our knowledge of the mechanisms that underlie myopia development remains limited, studies in mammalian models of high myopia have shown that active scleral remodeling plays a significant role in changes in eye size [9,10]. During the development of myopia, scleral collagen accumulation decreases[11,12] and its degradation increases[13,14], and these changes result in scleral dry weight loss[15]. After these changes, scleral collagen fibril diameter also decreases at the posterior pole of myopic eyes[9]. In addition to collagen alterations, proteoglycan synthesis in the sclera is also reduced in myopia[10,11]. As a result, the sclera progressively thins, and scleral creep rates increase[16,17], and these changes facilitate changes in eye size during the development of myopia[18]. Although changes in the scleral extracellular matrix during myopia development have been reported, the cellular and signaling factors that drive such changes have not been determined.

TGF-β has been reported to play a role in remodeling the scleral ECM in myopia. Three isoforms of TGF-β are down-regulated in myopia sclera and in vitro they increase collagen production of scleral fibroblasts[19]. Bone morphogenetic proteins (BMPs) are the largest subfamily of the transforming growth factor-β (TGF-β) superfamily; they are involved in numerous cellular functions, including development, cell proliferation, and ECM synthesis[20,21]. BMPs are essential for early eye morphogenesis[22] and may play a role in refractive error and the regulation of eye growth in myopia[23]. Genetic analyses have suggested that BMP2 is a myopia-related gene[24,25]. BMP-2 and bone morphogenetic protein receptors (BMPRs) are found in both human scleral fibroblasts and the human sclera[26]. In vitro, BMP-2 promotes cell proliferation and elicits changes in matrix metalloproteinase-2(MMP-2) and tissue inhibitor of metalloproteinase-2(TIMP-2)[27] expression, which influence collagen degeneration[28]. In addition, BMP-2 is among the most commonly used growth factor in chondrogenesis[29], and it induces chondrogenic differentiation in dermal fibroblasts and fibroblasts isolated from the inferior turbinate[29,30].

Our study sought to investigate the link between BMP-2 levels in the sclera and regulation scleral ECM. In particular, the study aimed to characterize the changes in BMP-2 expression in the sclera in a mammalian model of lens-induced myopia and recovery from myopia and to investigate the capacity of BMP-2 to regulate ECM production in fibroblasts in vitro.

## Materials and Methods

### Ethics Statement

All animals were treated according to the ARVO Statement for the Use of Animals in Ophthalmic and Vision Research, and this study was approved and monitored by the Institutional Animal Care and Use Committee of Zhongshan Ophthalmic Center (Permit Number: 2013–080). The guinea pigs were provided by the Shandong Traditional Chinese Medicine University. The animals were housed in an air-conditioned room with an ambient temperature of 16–26°C, a relative humidity of 40–70% and a 12-hour light-dark cycle with a daytime light intensity of approximately 500 lux. Pigmented guinea pigs were reared in opaque plastic boxes (465 mm long, 300 mm wide, and 180 mm deep) with wire mesh lids and provided with a commercial primate diet. In addition, fresh vegetables were provided twice daily, and water was freely available at all times. Animals were studied in adherence with the ARRIVE guidelines. All examinations were performed gently, and topical proparacaine HCl (Alcaine, 0.5%, Alcon Laboratories, Ft. Worth, TX) was used before measuring axial length. All efforts were made to minimize suffering.

### Preparation of lens-induced myopia in guinea pigs

Thirty three-week-old pigmented guinea pigs had myopia induced in their right eye using the defocus method with a concave lens (-4.00 D, poly methyl methacrylate (PMMA)); diameter, 11.5 mm; optical diameter, 11.35 mm; primitive arc, 7.5; King Tak & Jia Run Co. Beijing, China). The left eye in the same guinea pig served as the contralateral self-control. Guinea pigs were then randomly assigned to either a lens-induced myopia (LIM) group or a recovery group. Each group included 15 guinea pigs. The LIM group had a 3-week induction period wearing lenses, and the recovery group had a 3-week period with lenses and one week without lenses. Throughout the experiment, the lenses were cleaned at least once daily to prevent the form-deprival effect. Guinea pigs were examined using cycloplegic streak retinoscopy, and axial length was measured weekly by A-ultrasonic scanning.

### Primary cell culture and treatments

Healthy adult human eyes from donors (n = 3; ages 19, 22 and 25 years) were obtained from the Eye Bank of the Zhongshan Ophthalmic Center (Sun Yat-sen University). Our study was approved by the Ethics Committee of Sun Yat-sen University (Guangzhou, China), and complied with the tenets of the Declaration of Helsinki for biomedical research involving human subjects. The posterior scleral tissue was separated from the eyeball, cut into 1 mm × l mm pieces and placedin 25 mm^2^ plastic culture bottles in Dulbecco’s Modified Eagle Medium/F12 (DMEM/F12) containing 15% fetal bovine serum (FBS, Invitrogen Life Technologies, Carlsbad, CA, USA). They were incubated at 37°C in a humidified incubator containing 5% CO_2_. The growth medium was changed every three days. When the cells achieved a heavy primary monolayer, they were trypsinized for 2 min at room temperature in 0.25% trypsin/EDTA solution in phosphate buffered saline (PBS, Gibco) and then subcultured at a split ratio of 1:3 in a 25 mm^2^ plastic bottle(Corning Ltd, Lowell, MA). The fourth passage of cells was collected and treated with 0 ng, 10 ng/ml, 50 ng/ml, or 100 ng/ml recombinant human BMP-2 (rhBMP-2, R&D Systems, Minneapolis, MN, USA) or 10 ng/ml TGF-β1 (Peprotech Inc., Rocky Hill, NJ, USA) for three days, one week, or two weeks separately. The medium was changed every two to three days.

### Real-time Polymerase Chain Reaction (PCR)

Total RNA was isolated from cells with Trizol reagent (Invitrogen Corp., Carlsbad, CA, USA) according to the manufacturer's instructions. The cDNA was synthesized from 500 ng of total RNA (Takara Biotechnology, Japan). Real-time PCR was then performed using the Roche 480 real-time PCR system with LightCycler 480 SYBR Green I Master mix(Roche Diagnostics GmbH, Mannheim, Germany). The primers used are shown in Table 1. Gene expression was determined by the 2^- (ΔΔC (t))^ method using β-actin as the housekeeping gene.

**Table 1:** Primer sequences for real-time PCR.

Gene	Forward primers	Reverse primers	Product size (bp)	Accession No.
Β-actin	5'-CCAGAGGCGTACAGGGATAG-3'	5'-CCAACCGCGAGAAGATGA-3'-3'	97	NM_001101.3
COL1A1	5'-GTGCGATGACGTGATCTGTGA-3'	5'-CGGTGGTTTCTTGGTCGGT-3'	119	NM_000088
COL2A1	5'-AGACAGCATGACGCCGAG-3'	5'-GCGGATGCTCTCAATCTGGT-3'	87	BC007252
COL3A1	5'-GCCAAATATGTGTCTGTGACTCA-3'	5'-GGGCGAGTAGGAGCAGTTG-3'	145	NM_000090
SOX5	5'-CAAGGCAATCCAAGAAGCTC-3'	5'-CCAATCATTGCATGGCTAAA-3'	198	NM_006940
SOX6	5'-AGGATCTCGCTGGAAATCAA-3'	5'-CTGCCTCATCTCCTGTCTCC-3'	204	NM_001145819
SOX9	5'-AGCGAACGCACATCAAGAC-3'	5'-GCTGTAGTGTGGGAGGTTGAA-3'	110	NM_000346.3
RUNX2	5'-CCGCCTCAGTGATTTAGGGC-3'	5'-GGGTCTGTAATCTGACTCTGTCC-3	132	NM_001015051
HAPLN1	5'-TCTGGTGCTGATTTCAATCTGC-3'	5'-TGCTTGGATGTGAATAGCTCTG-3'	85	NM_001884
ACAN	5'-GTGCCTATCAGGACAAGGTCT-3'	5'-GATGCCTTTCACCACGACTTC-3'	167	NM_013227
PTHR1	5'-CTGGGCATGATTTACACCGTG-3'	5'-CAGTGCAGCCGCCTAAAGTA-3'	92	NM_001184744

### Western blot analysis

Guinea pigs were sacrificed with an intraperitoneal injection of 10% chloral hydrate (200 mg/kg), and their eyes were quickly enucleated. The anterior segment of the eye was cut away, and the posterior sclera was collected by removing the pigmental layers followed by grinding with liquid nitrogen. The total protein of the sclera or cultured cells was collected in ice-cold lysis buffer (50 mM Tris-HCl, pH 7.2, 150 mM NaCl, 2 mM EDTA, 10% (v/v) NP-40, 1 mM sodium orthovanadate, 50 mM sodium pyrophosphate, 100 mM sodium fluoride, 0.01% (v/v) aprotinin, 4 mg/ml of pepstatin A, 10 mg/ml of leupeptin, and 1 mM phenylmethylsulfonyl fluoride, PMSF) and incubated for 30 minutes. The lysates were homogenized and centrifuged at 12,000×g for 15 minutes. The supernatant was collected, and the total protein concentration was determined using the BCA protein assay kit (Beyotime, Shanghai, China). Then, 20 μg protein samples were separated by 10% SDS-PAGE and electrotransferred onto polyvinyl difluoride (PVDF) membranes (Millipore, Billerica, MA, USA) with 200 mA for two hours. Membranes were then blocked with 5% BSA (Sigma Chemical Co. St. Louis, MO, USA) for two hours at room temperature and subsequently incubated with anti-β-actin antibody (ab8226, 1:3,000 dilution; Abcam, San Francisco, CA, USA), anti-BMP-2 antibody (ab6285, 1:500 dilution; Abcam, San Francisco, CA, USA), anti-collagen I (ab34710, 1:1,000 dilution; Abcam), anti-collagen II (ab34712, 1:1,000 dilution; Abcam) or anti-collagen III antibody (ab7778, 1:5,000 dilution; Abcam), respectively, overnight at 4°C. Corresponding secondary antibodies were added (Boster Biological Technology, Wuhan, China) for 1 hour at room temperature. Proteins were detected using the enhanced chemiluminescence (ECL, Millipore, USA) detection system. β-actin served as an internal control, and the experiments were repeated at least three times.

### Immunofluorescence microscopy

Eyes were fixed in 4% neutral-buffered formalin for 24 h at 4°C. The posterior sclera surrounding the optic nerve was dehydrated and cut into frozen sections (5 μm thick). HSFs were sub-cultured in six-well chamber slides (Corning Ltd, Tokyo, Japan) and were treated with 0 ng/, 10 ng/ml, 50 ng/ml, or 100 ng/ml recombinant human BMP-2 or 10 ng/ml TGF-β1 for three days, one week, or two weeks separately. The samples were blocked with 10% normal goat serum (AR0009, Boster Biological Technology, Wuhan, China) for 30 minutes at room temperature. Subsequently, the frozen sections were incubated with BMP-2 antibody (1:500, Abcam), and the slides were incubated with anti-collagen I (1:1,000 dilution; Abcam), anti-collagen II (1:1,000 dilution; Abcam), and anti-collagen III antibodies (1:5,000 dilution; Abcam) to cell surface markers at 4°C overnight. The frozen sections were treated with with FITC-conjugated goat anti-mouse IgG antibodies (BA1101, 1:50 dilution, Boster Biological Technology, Wuhan, China) and the slides were then treated with FITC-conjugated goat anti-rabbit IgG antibodies (BA1105, 1:50 dilution, Boster Biological Technology) for one hour. Finally, the slides and frozen sections were mounted with mounting medium containing 4’,6-diamidino-2-phenylindole (DAPI, Roche Diagnostics, Indianapolis, IN, USA). The samples were observed using a confocal fluorescent microscope (LSM 510 META, Carl Zeiss, Jena, Germany).

### Toluidine blue staining assay

HSFs treated with BMP-2 and TGF-β1 for seven days were fixed in 4% paraformaldehyde and then stained with 0.05% toluidine blue (Sigma Chemical Co. St. Louis, MO, USA) for two hours. Subsequent washing steps were carried out in 25% ethanol and 90% ethanol to reduce unspecific binding of the dye. Images of the HSFs were taken with a light microscope.

### Analysis

Data were expressed as the mean±the standard error of the mean (SEM). The refractive power, axial length and BMP-2 expression data from each group of guinea pigs were analyzed using paired T-tests. Single-factor analysis of variance (ANOVA) was employed to determine the possible significant differences between the groups *in vitro*, followed by a post-hoc comparison using the Mann-Whitney U test when significance was detected (p<0.05). All data analyses were performed using SPSS (SPSS Inc., Chicago, IL, USA, version 19.0). P<0.05 was considered statistically significant.

## Results

### BMP-2 expression changes in the sclera of guinea pigs with lens-induced myopia (LIM) and during recovery from myopia

**Fig 1:**
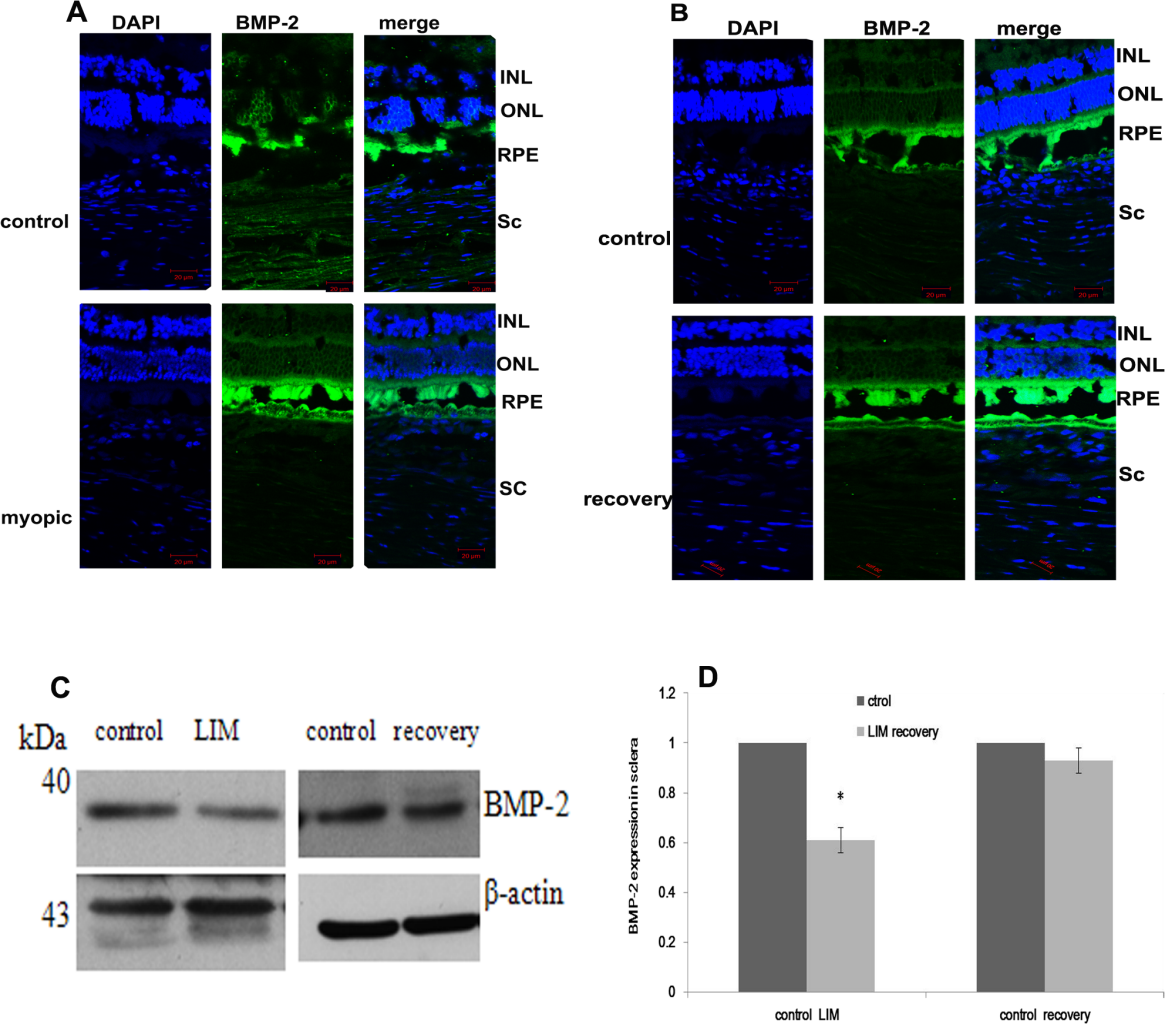
BMP-2 expression in myopia and recovery from myopia of guinea pigs. Immunofluorescence analysis of BMP-2 expression in the retina, RPE, choroid and sclera of guinea pigs. The staining in the myopic sclera of the LIM group was weaker than that of the control eyes (A). Changes in the expression of BMP-2 in the sclera of guinea pigs detected by western blot analysis (C,D). The data are expressed as the mean±SEM, * p≤0.05 vs. the contralateral eye. *INL* inner nuclear layer, *ONL* outer nuclear layer, *RPE* retinal pigmental epithelium, *chl* choroid, *Sc* sclera layer.

At the beginning of lens treatment, there were no significant differences in refraction or axial length of the eyes of the LIM group and the myopia recovery group (p>0.05). After three weeks of lens wear, the treated guinea pig eyes developed relative myopia (p<0.01, Table 2) and exhibited an elongated axial length (p<0.05, Table 3) compared to the contralateral control eyes. Refraction and axial length had regressed at one week after lens removal in the recovery group, and there were no differences in refraction (p = 0.14) or axial length (p = 0.87) between the treated and control eyes after recovery. Immunofluorescence staining of ocular sections revealed BMP-2 expression in the retina, retinal pigment epithelium, choroid and sclera of guinea pigs (Fig 1A and 1B). BMP-2 expression was significantly decreased in myopic sclera compared with the contralateral sclera in the LIM group (p = 0.01, Fig 1C and 1D). In the recovery group, BMP-2 expression was regained in the myopic eyes, and there was no distinction between the treated and contralateral eyes (p = 0.60, Fig 1C and 1D).

**Table 2:** The refractive power of the guinea pig eyes (D).

group	eye	0 day	3 weeks	4 weeks
LIM n = 15	right	3.18±0.329	0.65±0.457 *	
	left	3.625±0.266	2.95±0.343	
Recovery n = 15	right	3.95±0.404	0.85±0.441 *	2.63±0.362
	left	3.75±0.3436	3.08±0.454	3.13±0.341

Data are expressed as the mean±SEM

* p≤0.05 vs the contralateral eye.

**Table 3:** Axial lengths of the guinea pig eyes (mm).

group	eye	0 day	3 weeks	4 weeks
LIM n = 15	right	7.404±0.059	7.883±0.068 *	
	left	7.474±0.053	7.723±0.033	
Recovery n = 15	right	7.363±0.041	7.887±0.031 *	7.722±0.029#
	left	7.327±0.029	7.767±0.019	7.817±0.018

The data are expressed as the mean±SEM.

* p≤0.05 vs the contralateral eye.

### Collagen synthesis in HSFs treated with BMP-2 *in vitro*

**Fig 2:**
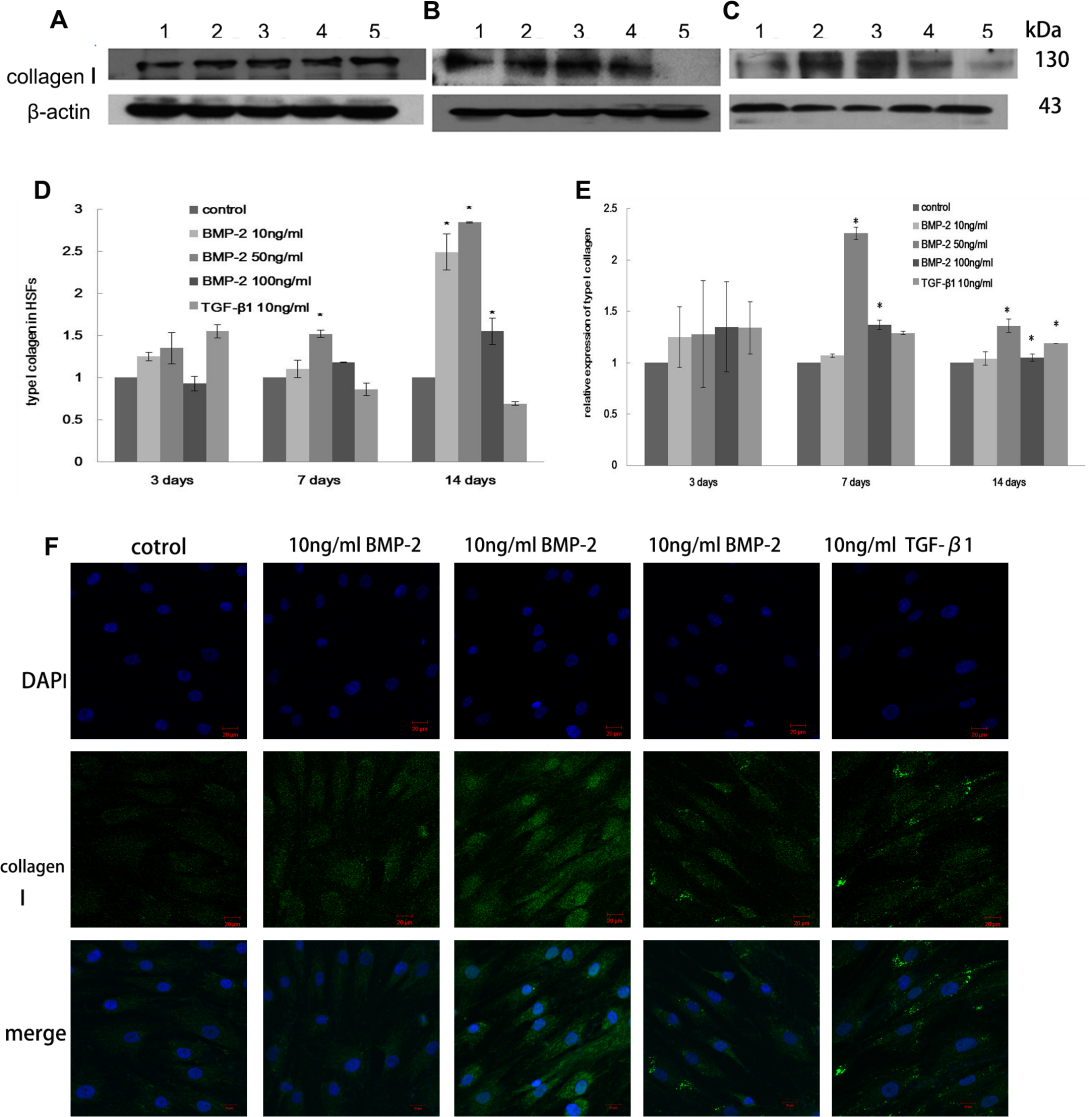
The effect of BMP-2 on type I collagen expression in HSFs. The synthesis of type I collagen in HSFs after 3 days (A), 1 week (B) and 2 weeks (C) of culture. Lane 1-Untreated control, Lane 2–10 ng/ml BMP-2, Lane 3- 50ng/ml BMP-2, Lane 4–100 ng/ml BMP-2, Lane 5- 10ng/ml TGF-β1. The data are expressed as the mean±SEM, N = 3, * p≤0.05 vs. control (D). The mRNA level of type I collagen in HSFs is shown (E). The data are expressed as the mean±SEM, N = 3, * p≤0.05 vs. control. Immunocytochemistry (F) also shows a BMP-2 induced increase in collagen type I expression in HSFs after 7 days culture.

**Fig 3:**
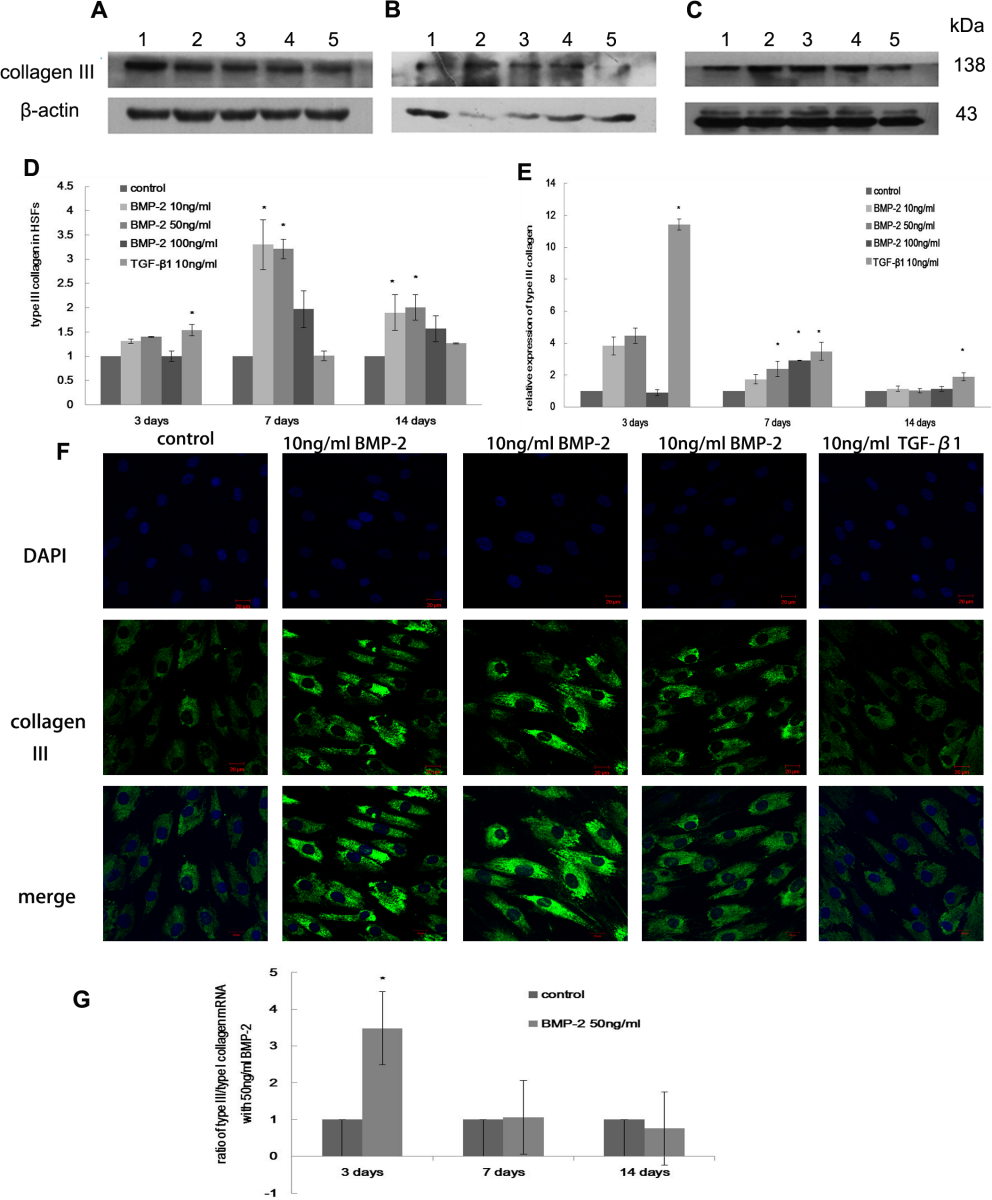
The effect of BMP-2 on type I collagen expression in HSFs. The expression of type III collagen in HSFs after 3 days (A), 1 week (B) and 2 weeks (C). Lane 1-Untreated control, Lane 2–10 ng/ml BMP-2, Lane 3–50 ng/ml BMP-2, Lane 4–100 ng/ml BMP-2, Lane 5–10 ng/ml TGF-β1. The data are expressed as the mean±SEM, N = 3, * p≤0.05 vs. control. Real-time PCR (E) also shows an increase in type III collagen after BMP-2 and TGF-β1 treatment (D). Immunocytochemistry (F) reveals the expression of type III collagen in HSFs after 7 days culture. The ratio of type III/I collagen mRNA increased significantly in HSFs treated with 50ng/BMP-2 for 3 days compared to control cells, but this ratio decreased and was not changed after 1 or 2 weeks of treatment (G). The data are expressed as the mean±SEM. N = 3, * p≤0.05 vs. control.

Type I collagen synthesis was increased 1.5 fold in HSFs cultured with 50 ng/ml BMP-2 for one week (p<0.05, Fig 2B). The mRNA level of type I collagen was elevated under treatment of 50 ng/ml, 100 ng/ml BMP-2 or 10 ng/ml TGF-β1 in culture compared with the control cells (p<0.05, Fig 2E). After two weeks, type I collagen protein synthesis was increased more than 2.5 fold by different concentrations of BMP-2 (p<0.05, Fig 2C). However, no changes were observed in the cells treated with BMP-2 after 3 days (p>0.05, Fig 2A). Type III collagen mRNA levels were increased in the presence of 50 ng/ml and 100 ng/ml BMP-2 in culture after seven days compared with the control cells (p<0.05, Fig 3E), but no differences were observed after either three days or two weeks (p>0.05). In contrast, type III collagen protein levels were significantly increased under treatment with 10 ng/ml or 50 ng/ml BMP-2 in the culture media for one or two weeks (p<0.05, Fig 3B and 3C). The stimulatory effect of 50 ng/ml BMP-2 on HSFs was evident after one week based on stronger immunostaining for type I and type III collagen (Figs 2F and 3F).

Furthermore, the ratio of type III/I collagen mRNA was increased significantly when cells were cultured with 50 ng/ml BMP-2 after three days (p<0.05), but this ratio did not differ from that in the control cells after one or two weeks (p>0.05, Fig 3G). Although TGF-β1 had no effect on the synthesis of type I collagen protein after three days, it stimulated the production of type III collagen compared with the control cells (p<0.05, Fig 3A). This result was consistent with the corresponding mRNA expression profiles obtained via real-time PCR (p<0.05, Fig 3E), and the effect on type III collagen mRNA was also observed after both one and two weeks (p<0.05). The effect of TGF-β1 on type I collagen mRNA appeared after both one and two weeks (p<0.05).

### Proteoglycan synthesis in HSFs

**Fig 4:**
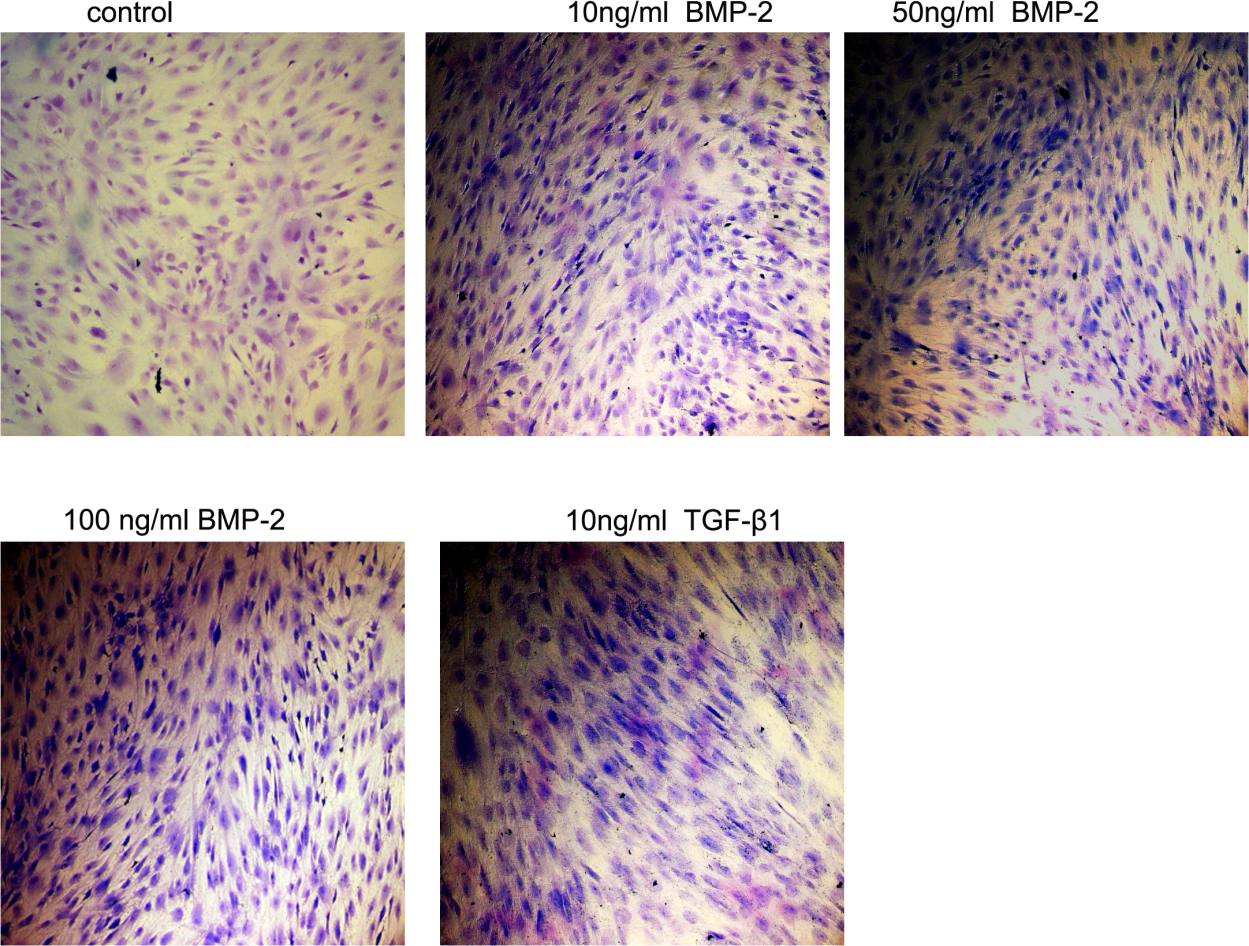
The effect of BMP-2 on GAG synthesis in HSFs. GAG synthesis was visualized by toluidine blue staining of HSFs in culture. GAG was observed after treatment with BMP-2 for one week but not in control cells. Original magnification: 40×.

**Fig 5:**
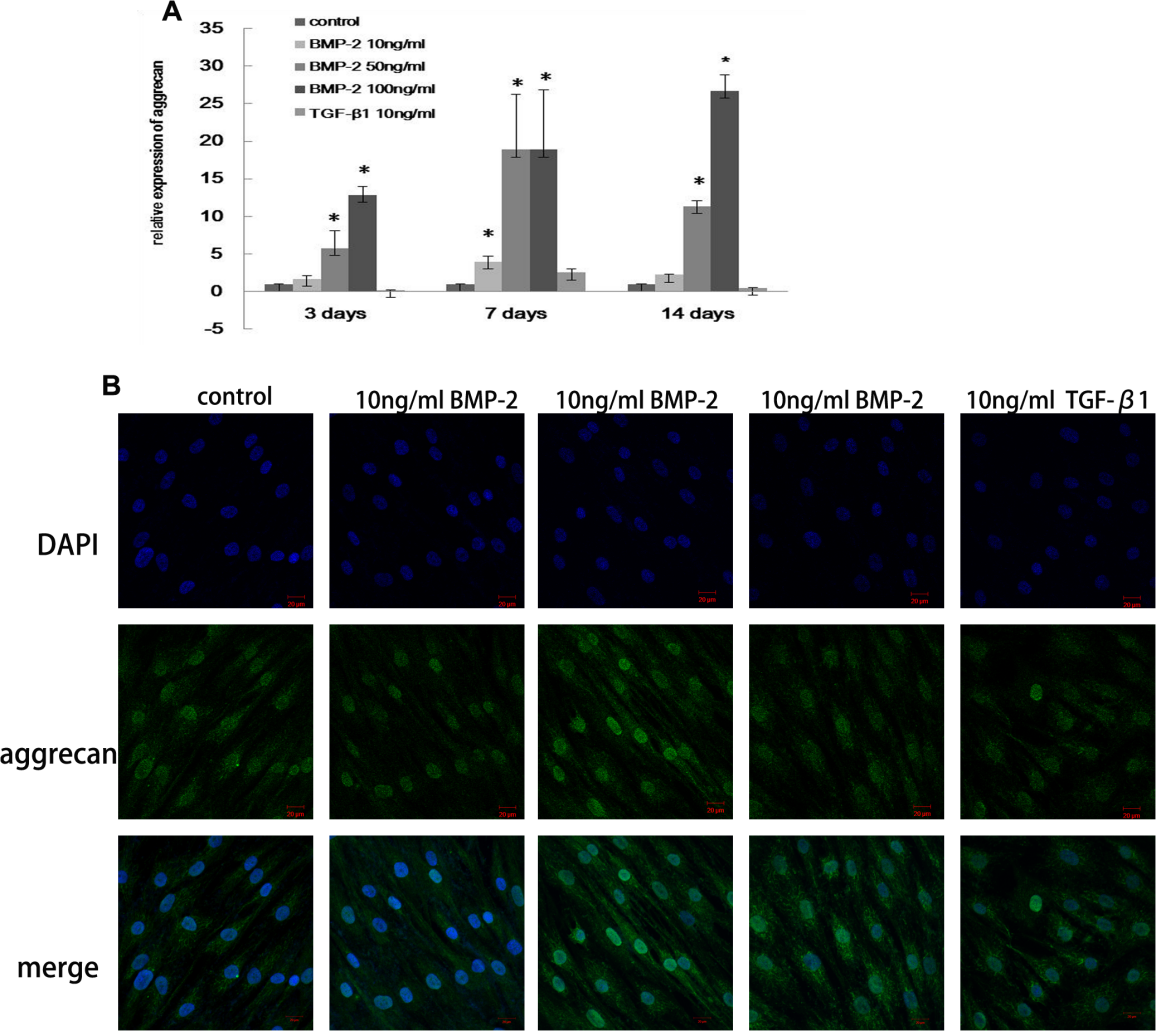
The effect of BMP-2 on aggrecan expression in HSFs. Aggrecan mRNA level in HSFs was analyzed after 3 days, 1 week and 2 weeks (A). Immunocytochemistry shows an increase in aggrecan expression after 7 days (B). The data are expressed as the mean±SEM. N = 3, * p≤0.05 vs. Control.

After treatment with BMP-2 for one week, HSFs exhibited positive metachromatic staining with toluidine blue, which demonstrates GAG synthesis (Fig 4). Aggrecan mRNA expression was increased 18.9-fold (p<0.05) in HSFs treated with 50 ng/ml or 100 ng/ml BMP-2 for one week (Fig 5A), and this effect was also apparent after two weeks. Immunostaining also revealed that aggrecan expression in HSFs was higher after culture with 50 ng/ml or 100 ng/ml BMP-2 for one week (Fig 5B).

### Cartilage-associated matrix and genes expression in HSFs in vitro

**Fig 6:**
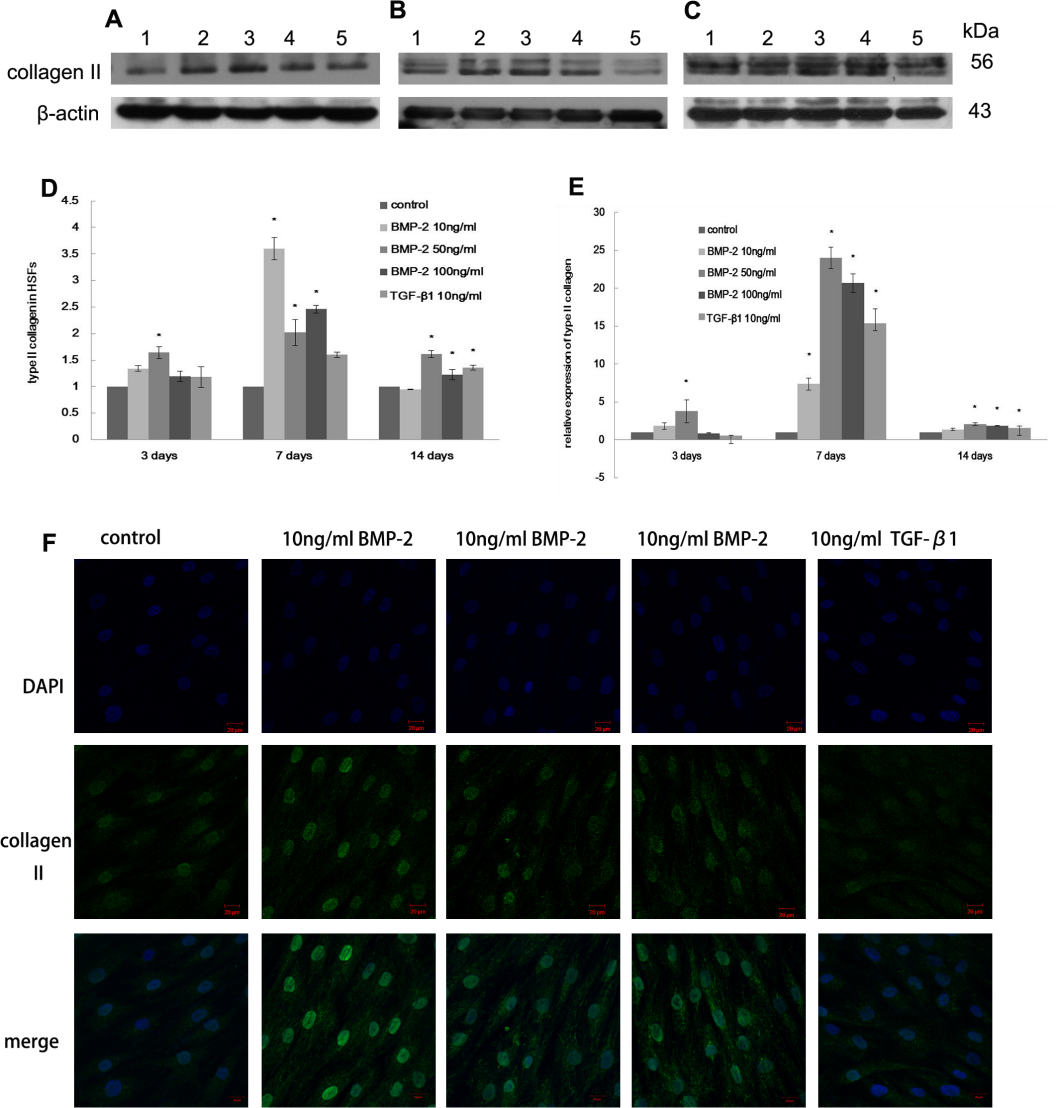
The effect of BMP-2 on type II collagen expression in HSFs. Type II collagen synthesis in HSFs after 3 days (A), 1 week (B), 2 weeks (C) detected by western lot analysis (D). Lane 1- Untreated control, Lane 2–10 ng/ml BMP-2, Lane 3–50 ng/ml BMP-2, Lane 4–100 ng/ml BMP-2, Lane 5–10 ng/ml TGF-β1. The expression of type II collagen mRNA was analyzed using real-time PCR (E). Immunocytochemistry showed an increase in collagen II expression in HSFs after 7 days of treatment with BMP-2 and TGF-β1 in culture (F). The data are expressed as the mean±SEM. N = 3, * p≤0.05 vs. Control.

Type II collagen protein, the primary marker of chondrogenic differentiation, was observed by immunostaining in HSFs cultured with BMP-2 for one week (Fig 6F). Western blot analysis showed that type II collagen protein was detectable in HSFs treated with 50 ng/ml BMP-2 for three days (p<0.05, Fig 6A) and was increased significantly in HSFs treated with 50 ng/ml or 100 ng/ml BMP-2 for one or two weeks compared to untreated cells (p<0.05, Fig 6B and 6C). No difference was observed between the 50 ng/ml and 100 ng/ml BMP-2 concentrations (p>0.05). Collagen II synthesis was also increased significantly in HSFs after treatment with 10 ng/ml TGF-β1 for two weeks (p = 0.01).

**Fig 7:**
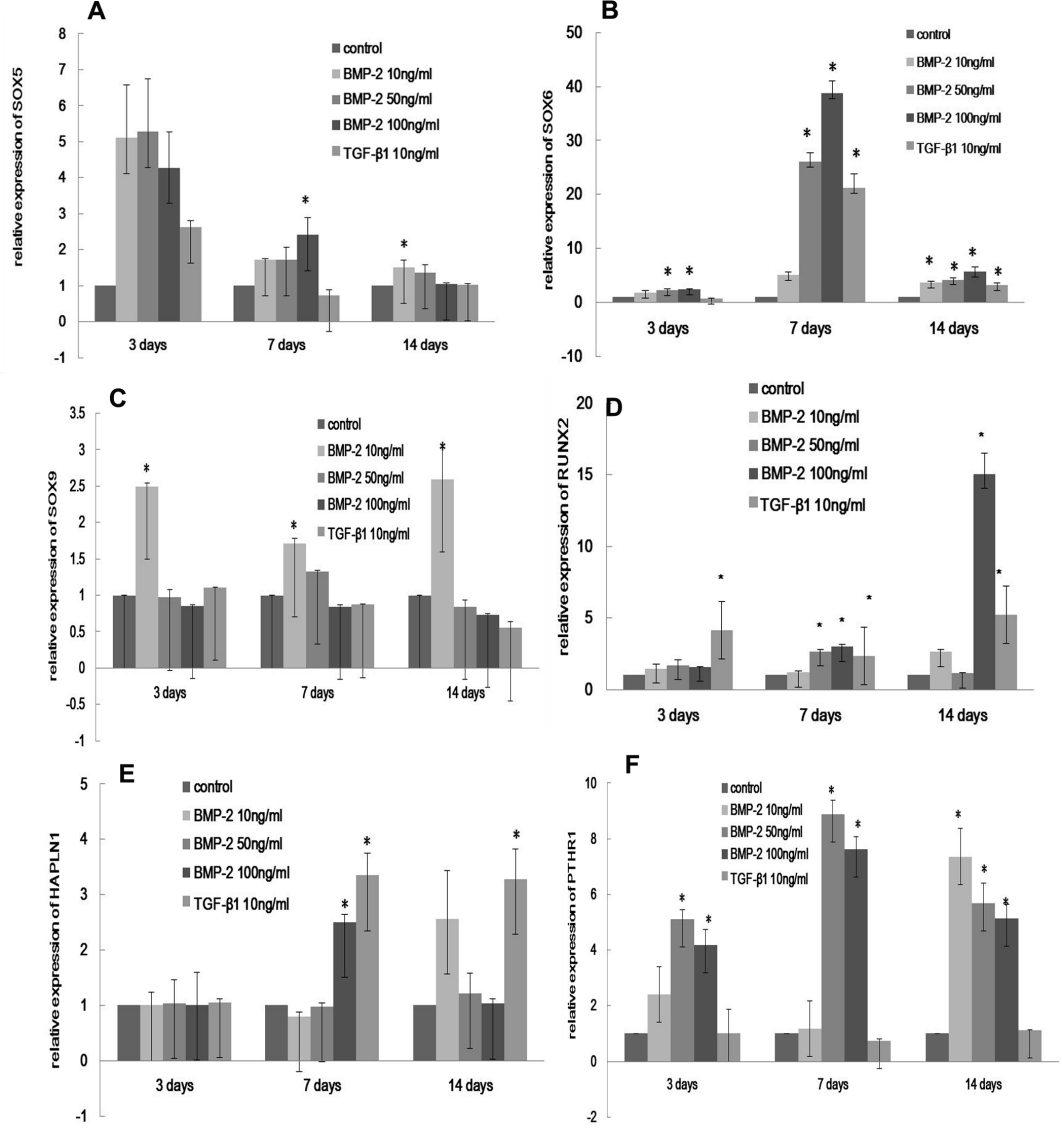
The effect of BMP-2 on chondrogenesis-related genes expression in HSFs. The mRNA level of SOX5 (A), SOX6 (B), SOX9 (C), RUNX2 (D), HAPLN (E), PTHR1 (F) was analyzed. HSFs were treated for 3 days, 1 week, and 2 weeks seperately. *: Denotes a significant difference relative to the control group, P< 0.05.

The expression of selected chondrogenesis genes, including SOX5, SOX6, SOX9, link-protein (HAPLN1), RUNX2, and PTHR1 was also measured, and increased mRNA expression of these genes was observed in HSFs treated with BMP-2 or TGF-β1 (Fig 7). After three days, significant increases in type II collagen, SOX6 and PTHR1 mRNA were detected in HSFs treated with either 50 ng/ml or 100 ng/ml BMP-2 (p<0.05, Figs 6E and 7B and 7F). Chondrogenesis was evident in cells cultured with BMP-2 for one week, and this effect was detectable throughout the following week. Type II collagen mRNA expression was increased more than 20 fold in HSFs treated with 50 ng/ml or 100 ng/ml BMP-2 for one week compared to untreated cells (p<0.05), but was only increased by approximately 2 fold after two weeks (Fig 6E). After one week, 50 ng/ml or 100 ng/ml BMP-2 induced the expression of the transcription factors RUNX2 (p<0.01, Fig 7D), SOX5 (p<0.05, Fig 7A), and HAPLN1 (p<0.01, Fig 7E) in HSFs. SOX9 mRNA expression increased continuously under treatment with 10 ng/ml BMP-2 for three days, one week, and two weeks (p<0.01, Fig 7C).

## Discussion

This study showed that BMP-2 was down-regulated in the sclera of lens-induced myopia guinea pig and that BMP-2 promoted the synthesis of ECM in HSFs in vitro. Therefore, these results support the hypothesis that reductions in BMP-2 expression during the development of myopia would lead to the down regulation of ECM synthesis during the development of myopia, as is observed in both animal and human myopia[11,31,32,33].

The myopia signals are transmitted from retina, through RPE and choroid (also gets transformed there), finally to the sclera[34]. BMP2 gene expression in chick RPE is regulated by a defocus signal[23], and in form-deprivation myopia, BMP-2 expression is reduced in the posterior sclera[35], but the BM2 gene expression in choroid of tree shrew is up-regulated during LIM[36,37]. Although the regulation direction in different tissues (RPE, choroid, sclera) of BMP-2 are different which may indicate their different roles in different tissues, it is evidential that BMP-2 signaling family is regulated in RPE, choroid and sclera, and is actively involved in the myopia and recovery signaling cascade. In our study, the BMP-2 expression profile in LIM was decreased and when the myopia regressed, its expression also recovered, so this provides further information about the possible role of BMP-2 in ocular growth and scleral remodeling in myopia. However, the potential involvement of BMP-2 in the signaling cascade that mediates sclera remodeling is unknown. Study has shown that levels of three TGF-β isoforms is decreased in the sclera of myopic eyes[19] and these alternation contribute to the major metabolic changes in collagen production, glycosaminoglycan synthesis and scleral biomechanics[15]. Therefore, we investigated the link of BMP-2 to the regulation of ECM synthesis in the sclera of eyes developing myopia.

BMP-2 stimulated type I and type III collagen synthesis in HSFs, with no change in the ratio of type III collagen to type I collagen mRNA after one or two weeks. In mammals, collagen accounts for as much as 90% of scleral dry weight, and the vast majority of this collagen is type I collagen[14]. Type I collagen levels are reduced in myopic eyes[31]. Furthermore, a significant reduction in the diameter of scleral collagen fibrils is observed in myopic eyes[9,33]. The change in fibril diameter is related to the ratio of fibrillar type III to type I collagen[38], and a higher ratio of type III/type I collagen probably results in the production of fibrils with a thinner diameter than when this ratio is lower[39]. Although the mRNA levels of collagen I and III were increased in HSFs treated with TGF-β1, their protein levels did not vary. These results showed that BMP-2 was more effective than TGF-β1 in stimulating HSFs to synthesize ECM.

BMP-2 also increased proteoglycan production in HSFs. Glycosaminoglycans (GAGs) are a small components of the scleral dry weight, and its levels regulate the biomechanical properties of the sclera[28] and control the rate of scleral creep by mediating the slippage of the lamellae across each other, hence contributing to the control of the axial elongation rate[40]. The negative charges of the GAG side chains sequester water, making the sclera nearly incompressible and contributing to its viscoelastic property[41,42]. Aggrecan is another important proteoglycan in scleral tissue; it is significantly modulated in the sclera during experimentally induced myopia and recovery, and changes in aggrecan may contribute to changes in the creep rate by facilitating or retarding the lateral slippage of lamellae in response to constant tensile force[43].

Because BMP-2 induces chondrogenic differentiation in fibroblasts[29,30], it may play a similar role in the sclera. After exposure to BMP-2, type II collagen production was increased, and cartilage-specific genes such as SOX5, SOX6, SOX9, RUNX2, HAPLN1 and PTHR1 contributing to extracellular matrix synthesis and chondrocyte differentiation[44,45,46] were upregulated. Previous work has suggested that the human sclera maintains chondrogenic potential throughout evolution[47], but the effect of chondrogenesis on scleral remodeling during myopia development is unclear. During the development of myopia, scleral changes in avians and mammals are often opposite in direction. For example, scleral GAG synthesis is found to be upregulated in birds and down-regulated in mammals developing myopia[48]. In chick and tree shrew, myopia is associated with the higher creep rate of sclera, and in myopic chick this change is accounted for the changes in the relative thickness of the cartilaginous[16]. We speculate that, the cartilage-associated matrix may play a completely different role in avian and mammalian myopia. The local organization of the cartilage regulate the biomechanical properties of the tissue[49]. Scleral cartilage is hypothesized to counter against the traction force of the extraocular muscle and against the accommodative force by intraocular muscles[47]. The chondrogenesis-associated ECM can generate a swelling pressure of several atmospheres that resists mechanical stress and tension[50].

In summary, this study demonstrates that scleral BMP-2 expression is reduced in myopia development and that BMP-2 may be involved in scleral ECM remodeling in myopia.

## Supporting Information

S1 ARRIVE Guidelines Checklist In the last column of the ARRIVE Guidelines Checklist I have loaded, I have filled it with section or paragraphs. The “title” and “abstract” means that in the title or abstract section of the manuscript. And the rest of vacancies filled with the numbers of paragraphs in the corresponding section.
(DOCX)
