# Supplementary material for: BMP-2 Is Involved in Scleral Remodeling in Myopia Development
Source: PLoS One. 2015 May 12;10(5):e0125219. doi: 10.1371/journal.pone.0125219 (PMC4429026; doi:10.1371/journal.pone.0125219)

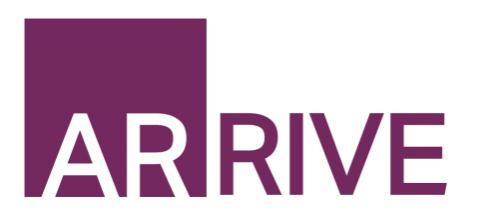


The ARRIVE Guidelines Checklist

Animal Research: Reporting In Vivo Experiments

Carol Kilkenny^1^, William J Browne^2^, Innes C Cuthill^3^, Michael Emerson^4^ and Douglas G Altman^5^

*^1^The National Centre for the Replacement, Refinement and Reduction of Animals in Research, London, UK, ^2^School of Veterinary Science, University of Bristol, Bristol, UK, ^3^School of Biological Sciences, University of Bristol, Bristol, UK, ^4^National Heart and Lung Institute, Imperial College London, UK, ^5^Centre for Statistics in Medicine, University of Oxford, Oxford, UK.*

|  | | ITEM | RECOMMENDATION | Section/ Paragraph |
| --- | --- | --- | --- | --- |
| 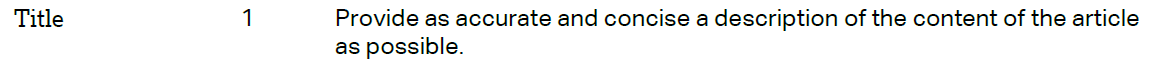 | | | Title |  |
| 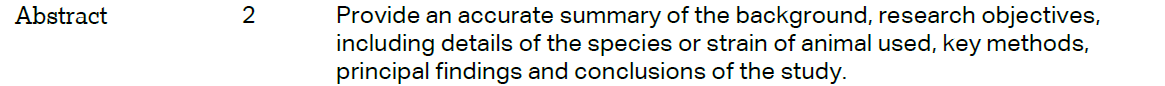 | | | Abstract |  |
| INTRODUCTION | | |  |  |
| 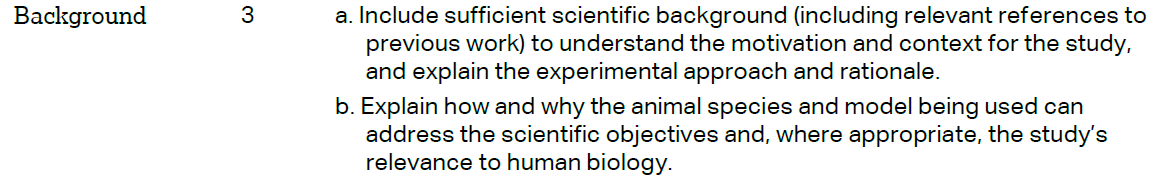 | | | Paragraph1,2  Paragraph 3 |  |
| 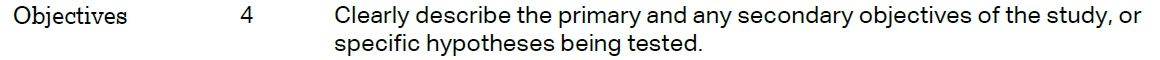 | | | Paragraph 3 |  |
| METHODS | | |  |  |
| 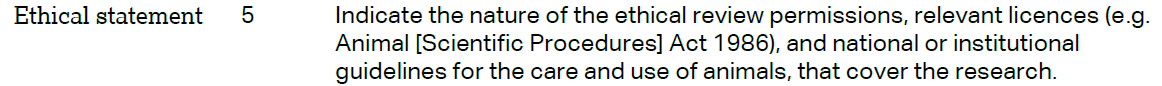 | | | Paragraph 1 |  |
| 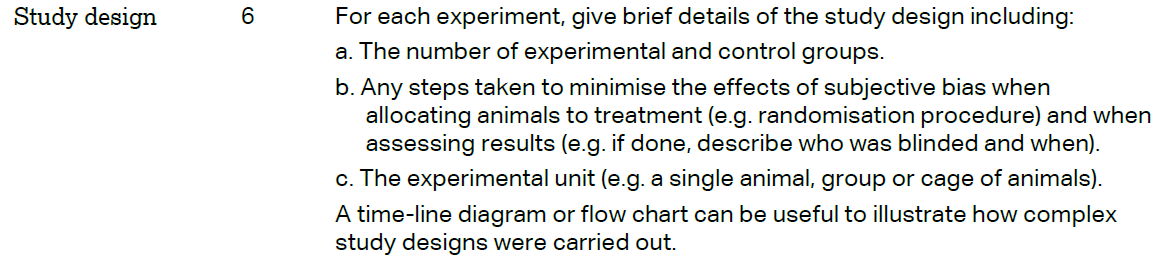 | | | Paragraph 1  Paragraph 2  Paragraph 2 |  |
| 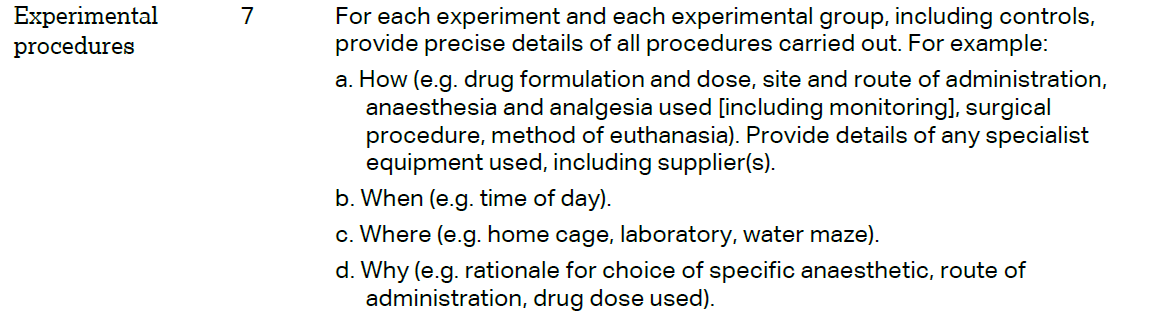 | | | Paragraph 4,5,6,7 |  |
| 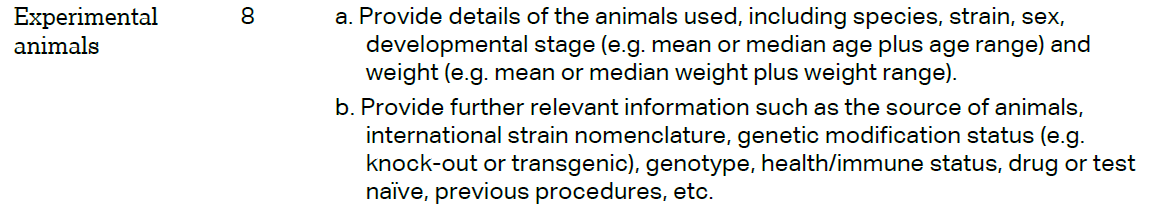 | | | Paragraph 1 |  |

The ARRIVE guidelines. Originally published in *PLoS Biology*, June 2010^1^

| 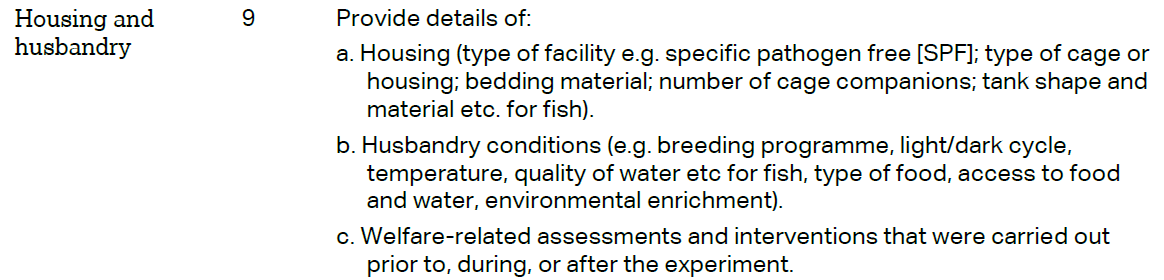 | Paragraph 1 | |
| --- | --- | --- |
| 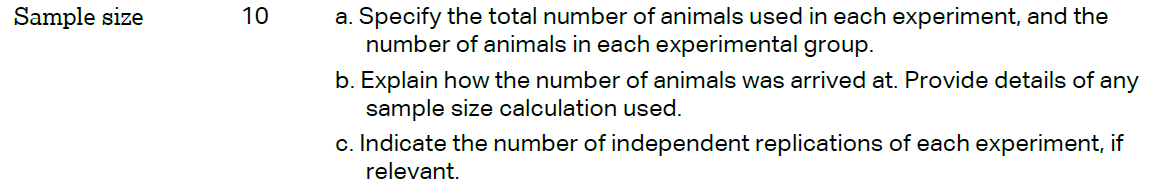 | Paragraph 2 | |
| 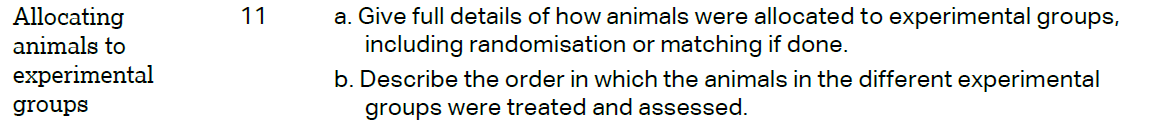 | Paragraph 2 | |
| 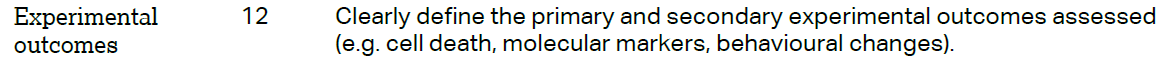 | Paragraph 3,5,6 | |
| 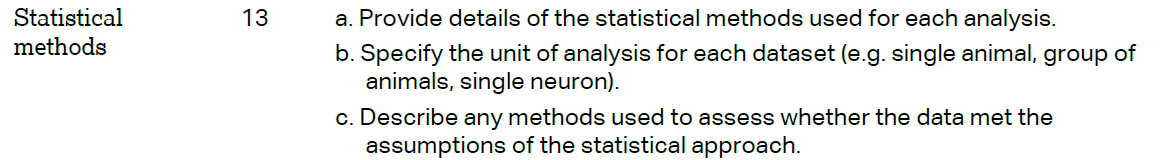 | Paragraph 7 | |
| RESULTS |  | |
| 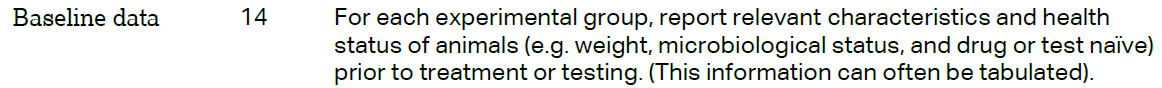 | Paragraph 1,Table 2,Table 3 | |
| 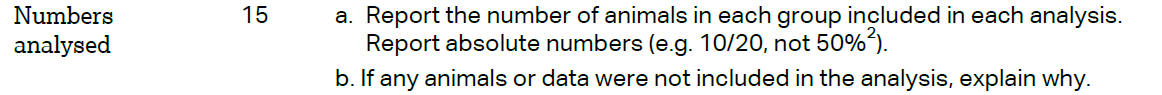 | Paragraph 1 | |
| 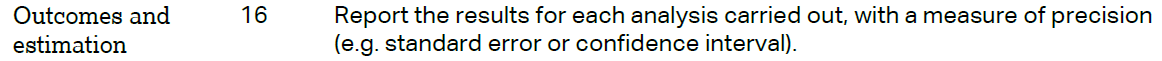 | Paragraph 2,3,4 | |
| 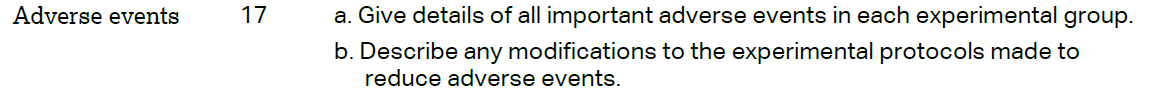 | Paragraph 2 | |
| DISCUSSION |  | |
| 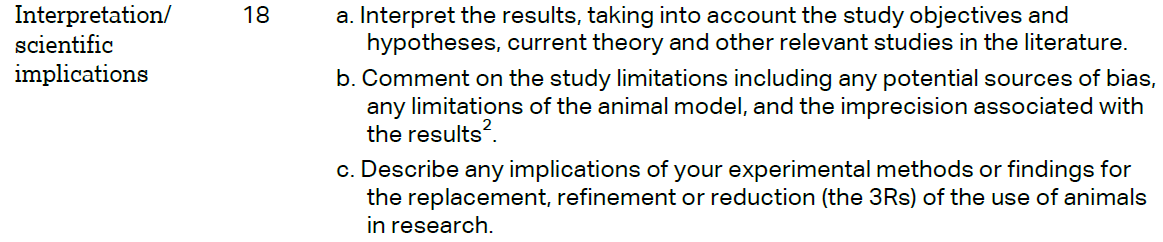 | Paragraph 1,2,3,4,5,6 | |
| 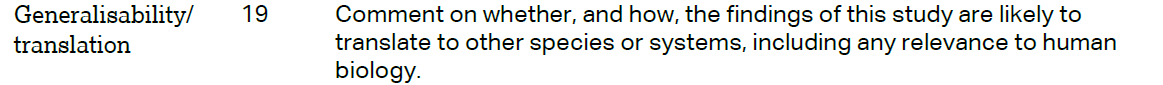 | Paragraph 1 | |
| 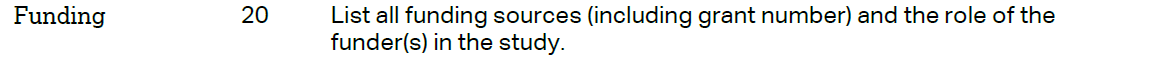 | | Paragraph 7 |


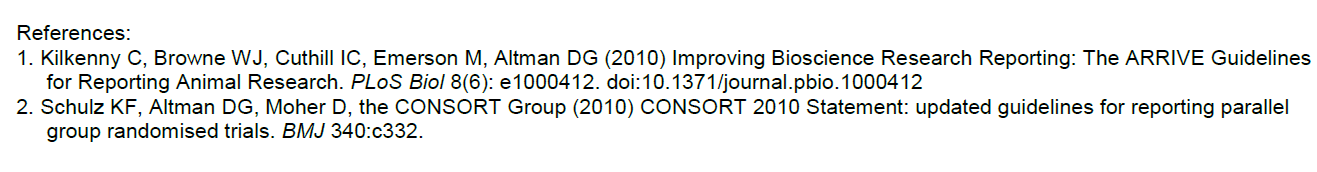

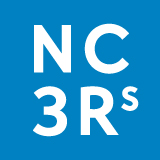

Supplement: S1 ARRIVE Guidelines Checklist — The “title” and “abstract” means that in the title or abstract section of the manuscript. And the rest of vacancies filled with the numbers of paragraphs in the corresponding section. (DOCX) [file pone.0125219.s001.docx]
